# Challenges and opportunities of telehealth digital equity to manage HIV and comorbidities for older persons living with HIV in New York State

**DOI:** 10.1186/s12913-022-08010-5

**Published:** 2022-05-06

**Authors:** Abigail Baim-Lance, Matthew Angulo, Mary Ann Chiasson, Helen-Maria Lekas, Rachel Schenkel, Jason Villarreal, Anyelina Cantos, Christine Kerr, Aarthi Nagaraja, Michael T. Yin, Peter Gordon

**Affiliations:** 1 Geriatric Research Education and Clinical Center (GRECC), James J Peters VA Medical Center, 130 W. Kingsbridge Rd, 4A-17, Bronx, VA 10468 USA; 2 Brookdale Department of Geriatrics and Palliative Medicine, Icahn School of Medicine at Mount Sinai, New York, NY USA; 3 Columbia University Mailman School of Public Health, New York, NY USA; 4 Division of Infectious Diseases, Columbia University Irving Medical Center, New York, NY USA; 5 Division of Social Solutions and Services Research, Nathan Kline Institute for Psychiatric Research, Orangeburg, NY USA; 6 Grossman School of Medicine, New York University, New York, NY USA; 7 Department of Family Medicine, Emory School of Medicine, Atlanta, GA USA; 8 Comprehensive Health Program, New York-Presbyterian Hospital, New York, NY USA; 9 Galileo Health, New York, NY USA; 10 Sun River Health, Peekskill, NY USA

**Keywords:** Telehealth, HIV and aging, Digital equity, Mixed methods, Access

## Abstract

**Background:**

Older persons living with HIV (PLWH) need routine healthcare to manage HIV and other comorbidities. This mixed methods study investigated digital equity, constituted as access, use and quality, of HIV and specialty telehealth services for PLWH > 50 years during the initial wave of the COVID-19 pandemic when services transitioned to remote care.

**Methods:**

A survey of closed and open-ended questions was administered to 80 English (*N* = 63) and Spanish (*N* = 17) speaking PLWH receiving HIV care at an Academic Medical Center (*N* = 50) or a Federally Qualified Health Center (*N* = 30) in New York State. Quantitative analyses examined characteristics predicting telehealth use and visit quality. Qualitative analyses utilized thematic coding to reveal common experiences. Results were integrated to deepen the interpretation.

**Results:**

Telehealth access and use were shaped by multiple related and unstable factors including devices and connectivity, technology literacy, and comfort including privacy concerns. Participants demonstrated their substantial effort to achieve the visit. The majority of patients with a telehealth visit perceived it as worse than an in-person visit by describing it as less interpersonal, and resulting in poorer outcomes, particularly participants with less formal education. Technology was not only a barrier to access, but also influenced perceptions of quality.

**Conclusions:**

In the COVID-19 pandemic initial wave, barriers to using telehealth were unequally distributed to those with more significant access and use challenges. Beyond these barriers, examining the components of equity indicate further challenges replicating in-person care using telehealth formats for older PLWH. Work remains to establish telehealth as both equitable and desirable for this population.

**Supplementary Information:**

The online version contains supplementary material available at 10.1186/s12913-022-08010-5.

## Background

People living with HIV (PLWH) in the United States are aging and experiencing a greater psychosocial and comorbidity burden compared to the general population [1–5]. To manage HIV and other comorbidities, older PLWH rely upon routine HIV primary and specialty healthcare services. Many of them receive care in Ryan White HIV/AIDS Programs, publicly funded comprehensive systems of HIV primary medical care, and essential support services including medication coverage for low-income patients [6]. In mid-March 2020, as COVID-19 began to surge in New York State and the “New York Pause” went into effect closing most face-to-face non-essential services [7], in-person outpatient HIV and specialty healthcare was substantially reduced or closed, and telehealth options rapidly expanded in an effort to maintain care while enabling necessary COVID-19 safety protocols.

Telehealth encompasses a spectrum of activities to deliver care remotely without direct physical contact including through synchronous video and telephone visits, asynchronous messaging, as well as remote monitoring [8]. Prior to COVID-19, studies of telehealth found it to be a promising means of care delivery [9–12] particularly in rural areas with few HIV providers and long travel distances to receive care [13]. Yet, broad adoption was sparse due to the reimbursement landscape and interpretation of ‘retention in care’ [11, 14] despite calls for its growth by some healthcare providers [15]. Telehealth expansion necessitated by the advent of COVID-19 has increased interest among many providers, administrators, researchers, and payers as a format for safe and effective HIV care delivery [14, 16, 17]. However, accounts of real-world implementation suggest challenges with uneven digital access [18], video technology [19], technology literacy, and making meaningful interpersonal connections [20]. Concerns have been captured in HIV and other populations requiring primary and specialty chronic care management [21, 22]. The unique stigma experiences of PLWH [23], pre-existing health and socioeconomic disparities and complex chronic disease management raise questions around the equity and effectiveness of telehealth as a medium of care for older PLWH. As calls to expand telehealth intensify, it is beneficial to assess early telehealth experiences among older PLWH during COVID-19.

### Conceptual framework

We sought to explore telehealth as an equitable means of delivering care to older PLWH drawing upon the constructs of access, use, and quality, which are identified in the literature as critical components to building digital equity [8, 24, 25] Access is defined as the potential to use healthcare services based on its availability including resource (e.g., device) availability on delivery and recipient sides. ‘Use’ connotes the actual delivery of a telehealth visit, a function of availability plus uptake or ‘demand.’ ‘Demand’ is largely framed in the literature as patients’ abilities with technology. Quality is the measure of an effective visit outcome to improve lives [8] or produce ‘good effects’ [25].

To consider questions related to telehealth equity, we developed and conducted a survey inclusive of open-ended questions with PLWH > 50 years receiving HIV care either in a large urban academic hospital-based medical center (AMC) in New York City, or a rural/semi-urban federally qualified health center (FQHC) in the Mid-Hudson Valley of New York State. Each program receives Ryan White funding to provide care to their majority publicly insured patients using a team-based, comprehensive medical and supportive services model. We also aimed to explore common and divergent experiences related to access, use and quality of telehealth across participants. To our knowledge there are no telehealth studies about older PLWH during the COVID-19 period using a multi-dimensional digital equity framework. Nor do we know of studies seeking to discern similarities and variations across a diverse, chronically ill population.

## Methods

### Program settings

Located in an urban academic hospital, the AMC HIV program is both inpatient and outpatient and provides HIV and primary care along with co-located mental health services and some specialty services. Many older PLWH who receive care at the AMC HIV program access specialty and inpatient care within the same facility. The program serves approximately 2400 patients, 42% of whom are > 50 years. In contrast, the FQHC’s HIV program is spread across several clinical locations in rural and peri-urban communities. Programs co-locate some specialties (mental health, dental, OB/GYN) but others require patients to seek care in the wider system. In two of the FQHC clinics of focus in this study, 240 HIV positive patients are cared for with 60% > 50 years. The AMC and FQHC are similar in that overall, 90% of their patients are virally suppressed. Both programs serve roughly the same percentage of Black patients (~ 40%) but the AMC serves a larger Latinx population (55% AMC compared to 21% FQHC) and a smaller white patient population (10% AMC compared to 39% FQHC).

In mid-March 2020, the HIV programs at the AMC and the FQHC largely transitioned to a telehealth care delivery model, though the AMC continued to see some patients in-person. Many community- and hospital-based specialty services used by PLWH at both sites converted to telehealth or temporarily closed except in emergency circumstances. Starting in July, the AMC and FQHC HIV programs began a fuller re-opening, with increased in-person care over the remainder of 2020.

### Sampling & recruitment

We randomly selected and made a list of 40% of each site’s census of HIV positive patients > 50 years of age. A member of the clinical or research team introduced the survey to patients on this list at a regularly scheduled health visit or by telephone. If interested, the patient was consented, and a research staff member administered the survey. At the AMC, out of 400 eligible participants on the list, approximately 170 were contacted, and of those 50 enrolled. At the AMC, out of 58 eligible participants, all were contacted and of those 30 enrolled. Common reasons for non-enrollment were: being unreachable by phone, disinterest, lack of time, and privacy concerns. Participants received a $25 ‘thank you’ for the time they devoted to the study. Approval for the study was granted by Columbia University Medical Center Institutional Review Board, IRB registration #00008612.

### Survey design & administration

The survey was developed by the study team based on clinical and content area expertise with some questions drawn from the Community Health Advisory & Information Network survey, a longitudinal study of representative samples of PLWH in New York City and the Tri-County region that began in 1996 [26] (see Additional File 1 for survey). The survey was administered by one of four trained study staff using Qualtrics, in English (3 staff conducted *N* = 63) or Spanish (1 staff conducted *N* = 17), in-person (*N* = 27) or via telephone (*N* = 53), between July and November 2020. Surveys were completed on a laptop or tablet by the study team member and, including the consent process, took between 30 and 60 minutes. Surveys were audio recorded for transcription and coding. English recordings were transcribed by three of the research staff directly into an a priori coding spreadsheet comprised of categories derived from the question areas in the survey (see questions and analysis for more details). Spanish language recordings were translated and transcribed by two of the study team members or a professional service, and content from the transcription was extracted and added to the coding spreadsheet.

### Closed and open-ended questions

Participants responded to a total of 44 open- and closed-ended questions (some with sub-questions applied with skip logic as applicable). We collected information in the following areas: 1) Physical and mental health history and current status; 2) COVID-19 infection history; 3) Overall management of HIV and other diseases during COVID-19; 4) Social networks, physical/social distancing and sources of support during COVID-19; and 5) Sociodemographic information and COVID-19 impact on resources. Questions were asked using a multi-select, Likert scale, and write-ins of specific answers (e.g., ‘how old are you today?’). Open-ended questions followed some closed questions to capture descriptions, meaning, and contexts of the standardized responses. Interviewers were encouraged to pick up on and further probe responses to clarify answers and elicit further detail. For example, after learning that a telehealth video or phone visit had occurred, participants were asked in an open manner what it was like to set up the visit, the quality of the interaction, the outcome of the visit, and any additional concerns. After asking about the quality of a telehealth visit using the Likert scale better/same/worse, interviewers would probe, ‘what made the visit better/same/worse?’ and followed up with additional questions based on the responses. The interview ended by asking participants to share ‘anything else important about [their] physical or mental health at the present time.’ Since surveys were recorded, unexpected explanations of close-ended questions to contextualize responses were also spontaneously captured and incorporated into the qualitative analysis.

### Analysis

For the quantitative analysis, responses were exported from Qualtrics to Microsoft Excel for preliminary descriptive summaries. R version 4.0.3 was used to conduct analyses. Closed ended questions were dichotomized and their responses aggregated to provide sufficient cell counts. For example, participant’s ease of technological use was determined by asking them to select challenges (if any) with technology. The list of available options included, “I don’t have technology for telehealth”, “I don’t know how to work the technology”, “I didn’t feel comfortable speaking with my provider by video/phone” or “I don’t have any challenges with technology”. To achieve sufficient cell counts, a participant that reported a challenge was categorized as *Yes* for difficulty using technology. The two outcomes of interest were use of telephone or video telehealth for HIV or specialty visits, and the reported quality of those visits (better/same/worse) compared to traditional in-person visits. We dichotomized the quality measure by combining better/same due to insufficient cell count and the study team’s specific interest in examining negative health experiences due to the telehealth format. Pearson’s chi-square test for independence was conducted to analyze all bivariate relationships of interest between specific variables with the outcomes. Variables of interest were selected a priori and derived from factors known in the literature to influence telehealth including physical components like age [27], degree of illness [28], being from a minority group, level of education, and socioeconomic status [11, 25]. Fisher’s Exact Test was used when expected cell counts were less than five. *P*-values < 0.05 were used to identify statistically significant associations.

The qualitative component used a framework method [29] in which a priori coding categories mapped to the five main areas of the survey. For example, for descriptions of ‘Overall Management of HIV and Other Diseases during COVID-19,’ coding categories (divided by HIV and other services) consisted of changes to visits, visit experiences, and telehealth comfort. In this process, text that supported these predicted codes also led to additional emergent codes (e.g., telehealth comfort vs technology comfort), iteratively expanding upon and updating the codebook to include relevant concepts. Reliability was assessed by the supervising coder who reviewed a subset of audio recordings/transcripts alongside the coded extractions. Analytic memos produced and discussed by the study team on use and quality of care revealed overall data patterns and outliers of experiences, which were supported by illustrative quotations.

Quantitative and qualitative analyses of the survey data were conducted in parallel using a convergent design in which independently arrived at findings pertaining to telehealth use and quality were then integrated [30]. Triangulating the two types of data helped in the development of more nuanced findings through the identification of compatibility, complementarity or divergence of findings and subsequently, explaining data patterns. For example, as we will describe, by bringing the data together we developed an explanation for how and why specific characteristic of PLWH > 50 years (e.g., level of formal education) might constrain telehealth use and opinions about quality.

## Results

Results are presented by the three domains that organized the study: ‘Telehealth Access,’ ‘Telehealth Use,’ and ‘Telehealth Quality.’ Statistically significant differences are followed by interpretative qualitative themes. Integrated results are displayed in Table 4.

### Sample description

Of the 80 participants, 62.5% (*N* = 50) received care from the AMC and 37.5% (*N* = 30) from the FQHC (Table 1). The sample consisted of 41 self-identified males, 38 females, and 1 transgender female. The median age was 59 (IR: 55–64). 21% (*N* = 17) of participants completed the survey in Spanish. 25% (*N* = 20) reported being born outside the United States or Puerto Rico, with 90% receiving care at the AMC. 74% (*N* = 59) of the participants identified as non-white (82% at the AMC vs 60% at the FQHC). 49% (*N* = 39) of participants had some education beyond a high school degree. The number of comorbidities other than HIV reported by participants ranged from 0 to 14 with an average of 4, and 54% (43) reported at least one mental health or substance use diagnosis. Behavioral health diagnoses along with neurology, cardiology, and rheumatology conditions were the most frequently reported. Compared to the AMC, participants at the FQHC were slightly older (60.4 vs 59 years) and had more comorbidities (80% vs. 46% had > 4). Just over half (53%, *N* = 42) of the overall sample reported having some type of difficulty using technology for telehealth.

**Table 1:** ‘Sociodemographic characteristics of study participants receiving care at one of two health centers

	*Academic Medical Center (AMC) n = 50*		*Federally Qualified Health Center (FQHC) n = 30*		*Total n = 80*	
	n	(%)	n	(%)	n	(%)
*Age*						
50 < 60	29	(58.0%)	14	(46.7%)	43	(53.8%)
60 ≤ 73	21	(42.0%)	16	(53.3%)	37	(46.2%)
*Gender*						
Male	26	(52.0%)	15	(50.0%)	41	(51.3%)
Female ^1^	24	(48.0%)	15	(50.0%)	39	(48.7%)
*Education*						
≤ High school	27	(54.0%)	14	(46.7%)	41	(51.3%)
> High school	23	(46.0%)	16	(53.3%)	39	(48.7%)
*Race*						
White	9	(18.0%)	12	(40.0%)	21	(26.3%)
Non-white ^2^	41	(82.0%)	18	(60.0%)	59	(73.7%)
*Language*						
English	33	(66.0%)	30	(100.0%)	63	(78.8%)
Spanish	17	(34.0%)	0	(0.0%)	17	(21.2%)
*Place of Birth*						
US and Puerto Rico	32	(64.0%)	28	(93.3%)	60	(75.0%)
Outside the US	18	(36.0%)	2	(6.7%)	20	(25.0%)
*Number of Comorbidities*						
0 < 4	27	(54.0%)	6	(20.0%)	33	(41.3%)
4 ≤ 14	23	(46.0%)	24	(80.0%)	47	(58.7%)
*Mental Health or Substance Use Condition*						
None	25	(50.0%)	12	(40.0%)	37	(46.2%)
At least one	25	(50.0%)	18	(60.0%)	43	(53.8%)
*Difficulties Using Technology*						
None	20	(40.0%)	18	(60.0%)	38	(47.5%)
Some	30	(60.0%)	12	(40.0%)	42	(52.5%)

^1^ For sufficient cell counts, one female transgender participant was categorized as Female

^2^ For sufficient cell counts, race was dichotomized given that no participants selected White and another race or ethnicity. Non-white encompasses participants who identified as Latinx, Black, and/or Native American

### Telehealth access and use

#### Demographic characteristics of telehealth users

Of the 80 participants, 73.8% (*N* = 59) had at least one telehealth interaction for any type of visit by video or telephone, and 26.2% (*N* = 21) did not. 53.8% (*N* = 43) had an HIV visit via telehealth, 38.8% (*N* = 31) had a specialty visit via telehealth, and 18.8% (*N* = 15) had both an HIV and specialty telehealth visit. Location of services was associated with having a telehealth visit with persons at the FQHC more likely to report having an HIV telehealth visit compared to persons receiving care at the AMC (*p*-value: 0.01). Location of services was also of borderline significance for whether participants received any telehealth visit (*p*-value:0.06). Among participants with an HIV telehealth visit, 60.5% (*N* = 26) had a visit by video while the remaining 39.5% (*N* = 17) had a visit via telephone. Participants reporting having a mental health condition or substance use were more likely to use telehealth, whether by video or telephone, compared to their peers (p-value: 0.04), though this was not the case for an HIV telehealth visit (Table 2).

**Table 2:** Telehealth use for any appointments and for HIV appointments among 80 study participants

	Have you had ***any***^*1*^ telehealth visits?					Have you had any ***HIV*** telehealth visits?				
	*Yes n=59*		*No n=21*		P-value	*Yes n=43*		*No n=37*		P-value
	n	(%)	n	(%)		n	(%)	n	(%)	
*Age*										
50 < 60	30	(50.8%)	13	(61.9%)	0.450	22	(51.2%)	21	(56.8%)	0.783
60 ≤ 73	29	(49.2%)	8	(38.1%)		21	(48.8%)	16	(43.2%)	
*Gender*										
Male	27	(45.8%)	14	(66.7%)	0.130	21	(48.8%)	20	(54.1%)	0.810
Female	32	(54.2%)	7	(33.3%)		22	(51.2%)	17	(46.0%)	
*Education*										
≤ High school	30	(50.8%)	11	(52.4%)	1	23	(53.5%)	18	(48.6%)	0.823
High school >	29	(49.2%)	10	(47.6%)		20	(46.5%)	19	(51.4%)	
*Race*										
Non-white	43	(72.9%)	16	(76.2%)	1	30	(69.8%)	29	(78.4%)	0.450
White	16	(27.1%)	5	(23.8%)		13	(30.2%)	8	(21.6%)	
*Language*										
English	47	(79.7%)	16	(76.2%)	0.761	36	(83.7%)	27	(73.0%)	0.370
Spanish	12	(20.3%)	5	(23.8%)		7	(16.3%)	10	(27.0%)	
*Place of Birth*										
US and Puerto Rico	44	(74.6%)	16	(76.2%)	0.178	34	(79.1%)	26	(70.3%)	0.441
Outside the US	15	(25.4%)	5	(23.8%)		9	(20.9%)	11	(29.7%)	
*Number of Comorbidities*										
0 < 4	21	(35.6%)	12	(57.1%)	0.121	14	(32.6%)	19	(51.4%)	0.113
4 ≤ 14	38	(64.4%)	9	(42.9%)		29	(67.4%)	18	(48.6%)	
*Mental Health or Substance Use Condition*										
None	23	(39.0%)	14	(66.7%)	**0.04**	16	(37.2%)	21	(56.8%)	0.115
At least one	36	(61.0%)	7	(33.3%)		27	(62.8%)	16	(43.2%)	
*Location of Services*										
AMC	33	(55.9%)	17	(81.0%)	0.06	21	(48.8%)	29	(78.4%)	**0.01**
FQHC	26	(44.1%)	4	(19.0%)		22	(51.2%)	8	(21.6%)	
*Difficulties Using Technology*										
None	29	(49.2%)	9	(42.9%)	0.799	22	(51.2%)	16	(43.2%)	0.509
Some	30	(50.8%)	12	(57.1%)		21	(48.8%)	21	(56.8%)	

^1^ Any encompasses a medical appointment for any of the following: cardiovascular disease, heart failure, cerebrovascular disease, seizure disorder, diabetes, hypertension, chronic kidney disease, liver failure, osteoporosis, arthritis, malignancies or cancers, substance use, hepatitis B, hepatitis C, surgical conditions, low back pain, or sciatica, peripheral neuropathy, dementia, or any other condition mentioned by participants.

#### Technology access limitations due to devices, telehealth apps, and connectivity

While our study defined telehealth as visits conducted by either video or telephone, participants focused their descriptions of access limitations on barriers to video visits. As one participant said, *“I can only do a phone, I can’t do anything”* (64 year old Black man at the FHQC). Some participants at both the AMC and the FQHC lacked devices like smart phones or laptops to take part in the video format (instead, some had visits by telephone, while others had no visits in the period). Others had devices but reported that they were of lower quality with minimal video functionality. One participant who had a smart phone said of the reason it would not work for telehealth, “*I think it’s cause my phone isn’t a computer like it has 4 gig of memory where I think like if you have an iPhone or Samsung – one thousand dollar phones – I think those phones would work better. But this is still like an ‘el cheapo’ … that’s what I suspect*” (56 year old white man at the AMC).

The problem of accessing and using video telehealth was more frequently expressed by participants at the AMC, who described specific challenges associated with using MyChart, the Electronic Medical Record application (MyChart App) needed to participate in a video visit in that setting. Some said they were unable to download the MyChart App due to their device’s limitations. Others could not make the video component work once it was installed. One participant with the MyChart App despite having used the platform for visits and test results discussed having to cancel a video visit with her social worker,

... *During the pandemic, I didn’t see, I didn’t have much communication with the [social worker]. Because the last time during the pandemic that I had an appointment with her, I tried to do by video, but when they called me for the video I couldn’t get to the video, so we had to cancel the appointment... when I got to that part that says you need to sign, from there, I couldn’t sign, I tried and I couldn’t, ever. And that was inconvenient”* (61 year old Latina).

Some compared challenges with MyChart to their ease of using other platforms (e.g., Whatsapp, Facetime) which they relied upon to keep in touch with family and friends particularly during COVID-19 physical distancing. This contrasted with the FQHC where the sign-in through another App was described as easy and “one-click,” and which also may have reduced the impact of the device limitations.

Participants across facilities also described basic broadband connectivity challenges. For those receiving care at the FQHC, the challenge was associated with rural access: “*I am in a [rural area so] I’m in a bit of a valley so getting WiFi … I didn’t have WiFi ability”* (56 year old white man). AMC participants implied challenges related to lacking in home-based internet.

#### Participant strategies and supportive Resources to overcome access limitations

Each limitation was accompanied by some participant descriptions of efforts to overcome them. For those without devices, a few said they borrowed them from families or partners, and in both healthcare settings some individuals described going to the clinic to use a computer to participate in an HIV or mental health visit. Some with devices of limited functionality for telehealth also described attempts to address the problem. For example, one AMC participant said he deleted items from his smart phone to create enough memory for the MyChart App. Several participants particularly at the AMC who described unstable or no internet connectivity where they lived sought out public connectivity points that are readily available in NYC to access primary and specialty telehealth video visits. A 63 year old Latina participant described attending a gastrointestinal specialty visit using a public access point called a “link box,” a New York City initiative to set up kiosks across the city to connect personal devices to free Wi-Fi:

Participant: *I told him look, I’m outside, I don’t have no internet so. He said don’t move we’re gonna work it through. It was a very difficult visit but we did it.*

Interviewer*:* You said it kept freezing?

Participant*: Cause it was outside. I had to go to the Link box. And so when I talked to him it was like on delay. And I told him I’m sorry, I don’t have the option to do Zoom and stuff like that [from home] at that time.*

In addition to describing efforts to access a video telehealth visit, the participant embedded in her story challenges with telehealth use once surmounting access obstacles. In the next section, we will describe other barriers when using telehealth, including the common experience of telehealth video defaulting to telephone during the visit.

Participants from both settings described receiving support from clinical staff, information technology specialists, friends and family to set up and use video telehealth. Some said this help was indispensable, while others noted that even with help they had to undertake several complicated steps. One participant at the AMC described speaking on a landline while setting up the App on his cell phone: “*She was talking me through the procedure on the house phone and I had the cell phone on the other hand, and she would ask me ‘well how’s it reading now,’ and I would tell her.”* (63 year old Latino). Others said they were unable to identify someone to help them; one participant at the FQHC said, “*I have a phone but no one to help use it right”* (66 year old white Native American man); another managed to use his smartphone for a video visit but described *frustrations* and having to “*fumble around … because I haven’t ever been shown how to do it.”* (70 year old Black man at the FQHC).

#### Neither savvy nor comfortable with technology/telehealth

Beyond technology and telehealth App access challenges, some participants including those who participated in a video visit said they lacked technology ‘*savvy*,’ a common idiom that was used along with describing oneself as ‘*computer illiterate.’* Lacking *savvy* was for some a general statement about limited technology abilities, attributed by some to being older by comparing their skills to their children or even the interviewer. For others, particularly those interfacing with the AMC’s more complex installation and log-on steps through the MyChart App, not being savvy was a reaction to lacking the competencies to set up and use video telehealth. Some described lacking the ability to troubleshoot arising technical problems already described, and new issues also arose as this account illustrates:

Participant: *I tried logging in just now to see how it was done, and they asked me for a zip code, the date of birth, just so many things.*

Interviewer*:* It’s too complicated?

Participant: *Way too much. Oh, and my email as well*. (59 year old Latina at the AMC).

The additional information required by the MyChart App, seemingly different from other technology platforms and Apps participants used (e.g., WhatsApp, Facetime), resulted in additional barriers to successfully using video telehealth.

Some participants particularly at the AMC also described their discomfort with and avoidance of telehealth as it related to concerns about loss of privacy. Telehealth visits (scheduled or impromptu phone calls) that came up when a participant was outside of a residential setting or with other people around could result in not participating. As one participant said, “*When I’m out on the street I don’t want to be talking with people around me you know*” (54 year old Black Native American man at the AMC). Another said, “*I don’t really like to use the phone for my medical conditions and all that because it’s too much people being in people’s privacy on their phones...I don’t want to participate in that”* (52 year old Black Latina at the AMC). Another participant echoed the sentiment but more specifically connected the visit to the possibility of being recorded saying, “*I didn’t want to talk about my life history on the phone. Some things are recorded. I wasn’t feeling that*” (52 year old Black woman at the AMC). Another participant took this further and described her concerns that information was not only recorded, but could also circulate without her control, “*Everything spreads so easily and is recorded, I am not very trustful*” (69 year old Latina at the AMC). The issue of privacy was not mentioned by the few participants who described finding public WiFi to enable their video visits.

#### Telehealth video defaulting to telephone

Overall, telephone visits were more common than video visits across the sample due to video access limitations described above. Telephones were also used when a video’s functionality failed and the visit “*got demoted to phone*” (53 year old Latina at the FQHC). One of the Spanish speaking participants said, “*The tech problems always ended up precluding being able to get video visits to work – [and we] always switched to phone*” (54 year old Latina at the AMC). Failed picture or sound might occur on either patient or provider sides. Again, some participants attributed this to the functional limits of less sophisticated (and costly) devices:

Participant: *I don’t have - I have an LGK51 - it’s an okay phone but I don’t think it’s - it’s not an Apple or a Samsung where it can process it fast enough like it’ll freeze up and then eventually after awhile we’ll just go over to regular telephone.*

Interviewer: So you tried the video?

Participant*:* “*Yeah it does work but then it’ll freeze up and stuff like that.*” (56 year old white man at the AMC).

Though the usual pattern was trying and failing at the video and then following up with a phone call, one participant described what he called a “crazy” hybrid video-phone neurology visit in which, “*We ended up talking on the phone while looking on the video. It was crazy I’m telling you. She said - let me call you so she called me and we were talking while we were looking at each other. We could hear each other but we couldn’t hear each other from the computer*” (66 Black man at the AMC).

### Telehealth quality

#### Demographic predictors of reported worse quality of an HIV telehealth visit

Participants were invited in the survey to compare telehealth to in-person care. 67.4% (*N* = 29) said the experience was worse compared to an in-person visit, 23.3% (*N* = 10) stated the telehealth visit was about the same, and 9.3% (*N* = 4) said it was better. Participants with a high school degree or fewer years of formal education had 4.75 (CI: 1.18–19.06) times higher odds of reporting a worse HIV telehealth experience compared to those with more formal education (*p*-value: 0.05). While not statistically significant, each of the seven participants who completed the survey in Spanish with an HIV telehealth visit reported the experience was worse than in person (Table 3).

**Table 3:** Comparison of HIV telehealth appointment quality to in-person appointments

	How did the HIV telehealth appointment compare to in-person?					
	*Worse n = 29*		*Same/Better n = 14*		*Total n = 43*	P-value
	n	(%)	n	(%)	n	
*Age*						
50 < 60	14	(48.3%)	8	(57.1%)	22	0.747
60 ≤ 73	15	(51.7%)	6	(42.9%)	21	
*Gender*						
Male	15	(51.7%)	6	(42.9%)	21	0.747
Female	14	(48.3%)	8	(57.1%)	22	
*Education*						
≤ High school	19	(65.5%)	4	(28.6%)	23	**0.048**
>High school	10	(34.5%	10	(71.4%)	20	
*Race*						
Non-white	22	(75.9%)	8	(57.1%)	30	0.292
White	7	(24.1%)	6	(42.9%)	13	
*Language*						
English	22	(75.9%)	14	(100.0%)	36	0.076
Spanish	7	(24.1%)	0	(0.0%)	7	
*Place of Birth*						
US and Puerto Rico	22	(75.9%)	12	(85.7%)	34	0.693
Outside the US	7	(24.1%)	2	(14.3%)	9	
*Number of Comorbidities*						
0 < 4	10	(34.5%)	4	(28.6%)	14	1
4 ≤ 14	19	(65.5%)	10	(71.4%)	29	
*Mental Health or Substance Use Condition*						
None	12	(41.4%)	4	(28.6%)	16	0.512
At least one	17	(58.6%)	10	(71.4%)	27	
*Location of Services*						
AMC	16	(55.2%)	5	(35.7%)	21	0.332
FQHC	13	(44.8%)	9	(64.3%)	22	
*Difficulties Using Technology*						
None	13	(44.8%)	9	(64.3%)	22	0.332
Some	16	(55.2%)	5	(35.7%)	21	
*Telehealth Format*						
Phone	13	(44.8%)	4	(28.6%)	17	0.343
Video	16	(55.2%)	10	(71.4%)	26	

#### Reasons for “worse” telehealth experiences

Technology overwhelms the visit: Technological limits to telehealth outlined above as they related to access and use also permeated and shaped the quality of the telehealth visit. The negative influence of experiencing technology glitches and the default of video to telephone resulted in experiencing telehealth as a *nuisance, frustration,* and *difficulty.* Pointing out the irony of the impact on a specialty therapy visit, one participant said “*The audio is so bad that the conversation connection is cut. Do you understand? It is a therapy and you have to repeat what you said. It is a nuisance, I don’t like it. That is because my internet is bad. I told her I’d rather come in*” (55 year old Latina at the AMC). Technology also influenced the way participants practiced the visit. On the lighter end in reference to the hand-holding nature of a smartphone device, a participant explained “*you gotta figure out how to hold things, face contact … it’s just odd*” (62 year old white woman from the FQHC). A more concerning reaction to therapy by a few participants was the lack of comfort sharing deeper feelings outside of the face-to-face encounter. One participant said that sharing deeply would be “too traumatizing” (52 year old Black woman). Another Spanish speaking participant said her fear that telehealth visits were recorded inhibited her from talking. She demonstrated her point by saying, “*If something has happened to me, I say, ‘no, I’m okay, nothing happened … everything is fine.’*” (55 year old Latina at the AMC).

What is missed in virtual interactions without in-person care: While in the closed response most participants rated the telehealth visit as worse than the in-person, many of those same individuals later described the interaction as going well particularly when they already had an established relationship with providers, and the goals of communication were achieved. These accounts emphasized the ability to talk in the same manner as in-person about “*things bothering me physically, mentally, emotionally*” (63 year old Black woman at the AMC). Yet, time and again, even when visits went well, participants also pointed out that they were “*not the same as in person*” (61 year old white man at the FQHC). Telehealth lacked *laying* or *putting hands* during the medical examination, the taking of vitals, and the ability to show a doctor something and for them to *see, feel, and touch* which rendered the care less comprehensive and *personal* (52 year old Black Latina at the AMC). The importance of reading the body went both ways; participants ‘read’ their providers as much as they were being read. As one participant said, “*I like looking into their eyes”* (64 year old Black man at the FQHC).

Further, telehealth encounters were described as more focused on specific topics and patient-provider conversations were less exploratory and open ended compared to in-person visits. Referring to how conversations naturally unfold during in-person care, a participant described it as a “*chain reaction, cause you talk about one thing and then you remember … you started talking about medications and different things in person …*” while in contrast, “*you wanted to say [things] over the phone and then you didn’t remember*” (60 year old Black man at the AMC). Others also agreed that memory was both important but hampered in the virtual compared to the in-person visit. A Spanish speaking participant at the AMC said of the in-person visit, “*we have more time, I would say, more ease to express yourself better than by video because sometimes with the video with whatever little interruption, you lose or forget what you were going to ask or what you wanted to tell him*” (56 year old Latina). Along with remembering and interacting, the amount of time spent with the provider also emerged as significant. Some participants felt that the in-person visits were longer than telehealth visits. The importance of time can be inferred in the description of another Spanish language participant about her deliberate back and forth with her language-discordant nutritionist: “*when she speaks English, she speaks, a Spanglish, between Spanish and English, and she speaks slowly, so I can get her, and she gets me, because slow like that we get each other … we try to find a way to understand each other, and if we have to repeat, and spell it out, she does that and so do I”* (55 year old Latina at the AMC). This back and forth may have occurred by telehealth, though for some it did not feel possible in that format.

Experiences of subpar outcomes*:* Some participants believed that telehealth resulted in subpar care outcomes. Participants described the quality of follow-up communication and care coordination as worse in the context of telehealth. One participant felt anxious about not hearing about test results, while another said telehealth and the pandemic more broadly (by overworking staff or allowing remote work) contributed to his “*slipping through the cracks”* (64 Black man at the FQHC) and a delay in being referred to much-needed follow up eye care. A third participant at the AMC described delayed scheduling of speech therapy after a stroke. Another category of concern was a more general feeling of being dissatisfied with the clinical outcome. A Spanish speaking participant went so far as to say a neurology visit was ‘*useless’* due to a cursory physical video exam (69 year old Latina). Another AMC participant explained why telehealth did not meet his needs: “*The one time I did the video thing I don’t think anything conveyed, I just have to say no, I’m not comfortable with that, none by telehealth”* (57 year old white man). A final category reported by participants was that video or telephone telehealth resulted in actual referral errors. An extreme illustration was a Spanish speaking participant whose neurologist ordered a referral for physical therapy on the wrong side of the body,

*When I had an appointment by video, I don’t know, there was a misunderstanding, because I was telling him about my right leg, my right knee, but he focused on the left, I don’t know why. Well maybe it’s that he has treated my left side previously but the problem was on my right side for this appointment, and he sent me for a referral [for physical therapy] for the left leg … So it was like … we didn’t understand each other* via *the camera, or he misunderstood, looking at my old record or I don’t know what happened, so I didn’t like the video visit … you know sometimes when you are talking by the video things are faster* (56 year old Latina).

#### Reasons for “Better or Same” telehealth experiences

Convenience and nothing lost (for now): Some participants who did not have access and use barriers said they preferred telehealth visits some or all of the time because it was easier logistically, less time consuming, minimized in-office wait times, and some participants with good connectivity and privacy in their residential spaces felt more comfortable taking a medical visit there compared to the clinic. Another positive quality of the video visit was having the full attention of the provider. Some AMC participants using the MyChart App (even those with negative feelings about a virtual medical visit) also liked being able to get in touch with their providers using asynchronous texting, checking results, and scheduling visits. It was not uncommon for participants to couch their acceptance of telehealth as conditional on factors like their health status (being not too sick) and perhaps most importantly COVID-19, graphically illustrated by one participant who said, “*with COVID, it is fine but like my next one is going to doctors”* (64 year old Black woman at the FQHC).

Participants overall expressed COVID-19 vulnerability as a result of HIV infection with comments like, “*if I get COVID-19 I will go on a ventilator and die*” (61 year old white man at the FQHC). For these participants, averting COVID-19 exposure translated into at least temporary acceptance of receiving care through telehealth. See Table 4 for integrated quantitative and qualitative findings.

**Table 4:** Joint Display of Converging Quantitative and Qualitative Findings

	QUANTITATIVE	QUALITATIVE
**Overall Telehealth Access and Use**	Approximately three-fourths of participants engaged in any telehealth and half of participants in an HIV video or telephone visit after the COVID-19 pandemic began	A range of telehealth access challenges include no or unstable: - Devices - WiFi connectivity - User unfriendly telehealth App (specific to AMC)
	Approximately half of participants reported having difficulty with technology	Participants reported lacking competency (‘savvy’) with technology and/or telehealth
	For HIV visits, 60% by video and 40% by telephone	Common for video to ‘default to phone’ during the visit
		Participants mobilized to overcome challenges with access and use, inconsistently resulting in a visit
		Concerns about technology/telehealth privacy
**Differences in Telehealth Use by Significant Characteristics**	***Location of Service*:** FQHC more likely to have HIV telehealth visit (and more likely to have any telehealth visit) than at the AMC	FQHC use of single sign-on system to log into a telehealth visit much simpler compared to AMC multi-step log-in and connection within ‘My Chart’ App
	***Mental Health or Substance Use Diagnosis****:* Participants with mental health or substance use diagnoses more likely to have any telehealth visit compared to no diagnosis.	Telehealth favors ‘talk’ over physical diagnostic and care elements, which may have allowed for maintenance of therapy visits; *however, see ‘influences upon ‘worse’ for caveats*
**Overall HIV Telehealth Quality**	70% of participants reported that telehealth was ‘worse’ than in-person visits	Reasons for rating telehealth as ‘worse’: Less time with provider, increased forgetfulness, limited interpersonal physical interactions shaping quality including outcomes, altered communication, self-censoring Technology challenges worsened the visit when it occurred Ineffective outcomes defined as: no follow-up, referral error, generally ‘useless’ exchange
	30% reported telehealth was same/better than in-person visits	Reasons for rating telehealth as ‘same/better’: more efficient transport to visit, less waiting, ‘same result’ [e.g., effective]
		Reporting ‘worse’ quality did not mean unacceptable visit when recounting details if relationship established, health status stable, and outcomes are ‘same result’
**Differences in Reported Telehealth Quality by Significant Characteristics**	***Education****:* Participants with fewer years of formal education more likely to report worse quality	*See* Reasons related to a worse telehealth visit; intensified combination of technology challenges and loss of interpersonal communication and interactions
	***Language of Survey:*** All Spanish language participants reported worse quality (*N* = 7)	*See* Reasons related to a worse telehealth visit; less time and in-person physical exchange may negatively impact care with language-discordant providers

## Discussion

Overall, almost three-fourths of study participants had at least one telehealth visit by video or telephone for an HIV or specialty visit, and half had an HIV visit between the March 2020 New York State Pause and the time of the survey. Roughly 40% of these visits occurred by telephone rather than video. Based solely on measuring use, participants overall appeared to be relatively connected to care by either telephone or video during the period, which is consistent with other findings of HIV care in the US context during COVID-19 [19, 31] Further, a mental health or substance use diagnosis was a significant predictor of a telehealth visit for a non-HIV visit, a finding that may indicate that some participants with these diagnoses in our sample maintained their routine therapy.

Combining quantitative results with qualitative themes provided a deepened and more complex picture of telehealth challenges, including the types of challenges and how they negatively affected telehealth equity. Many of the participants lacked the necessary resources to engage in telehealth, particularly the video delivery format. Receiving care at the FQHC was a significant predictor of whether a visit occurred. We attributed this to the fact that use of video telehealth was harder at the AMC where access and use of the MyChart App proved onerous, compounded by the need for high end communication devices (e.g., smart phones or laptops), consistent functionality and the know-how to navigate the platform. The FQHC’s one-click log-in and use required some video stability but seemed to be operational with less sophisticated device and participant technology savvy. Telehealth support, a feature advocated in the literature [9], facilitated access and use at both locations, but it could not entirely overcome the significant resource barriers. Moreover, even with support some technology know-how was needed by the participant, as suggested by the individual who received help on a landline while installing the technology on a portable device.

The demanding nature of the App shows how, under certain circumstances, the delivery of digital care is not only potentially underpinned by existing health disparities but those disparities can widen in the digital sphere [8, 20, 32]. This widening is shaped by barriers in access to and use of devices, connectivity technology, and Apps. Findings further describe how the unpredictable nature of telehealth components exacerbate disparities when existing resources were inconsistently operational, graphically illustrated by video technology that worked in one moment but failed in the next. Participants in this context then needed to engage in attempts - with support from their HIV programs - to both secure the necessary resources (i.e., have/find/borrow a phone or internet terminal) and then mobilize them towards being functionally sufficient for telehealth (i.e., clear out memory, seek out a public WiFi point).

Time and again, participants demonstrated adapting to new telehealth ‘interaction chains’ [33] to: secure multiple kinds of resources (each with the built-in possibility of failing); mobilize them towards a successful visit (their successful alignment also uncertain); and apply know-how to address routine and emergent complications. Given these conditions, it is not hard to infer how some participants had missed or substandard visits, as much as ones that occurred and felt of reasonable quality. As telehealth expands the potential for access, our findings suggest that it can concomitantly create – or intensify – the makings of a fragile environment for some patients as well as additional work for them to acquire and mobilize necessary resources for routine care.

Findings also point to distinguishing participants’ degree of comfort with technology from comfort with telehealth, both important features of ‘telehealth demand.’ Participants across urban and rural settings put specific language (‘lack of savvy,’ ‘illiterate’) to lacking the skills to navigating technology, particularly as challenges arose. The need for better support, training and coaching to use technology have been widely identified to increase the effective use of telehealth [8, 19, 33]. The analysis further suggests the value of designing coaching to assist patients with real-time point of use problem-solving. Wootton et al [34] similarly suggest using ongoing text messaging to accompany a patient’s visit to address challenges arising during the entirety of a visit. Further, our findings point out the separate and important influence of telehealth discomfort due to privacy concerns. This issue has been identified in telehealth services involving PLWH due to HIV disclosure fears [35], as well as in other ‘remote’ health programs such as mobile clinics where, by moving beyond clinic walls, patients raise concerns about inadvertent disclosure of HIV and other health-related information [36].

Overall, of those who completed a visit, nearly 70% felt it was worse compared to their usual in-person visits, a departure from several studies prior to [13, 37] and during [31, 32] the pandemic that identified patient perceptions of telehealth as equal or better to in-person care. This may be due to our framing telehealth in comparison to in-person care; in so doing and in explaining what was worse, several insights about the features of telehealth emerged. The first and overarching point was the influence of technology not only on access and use, but also on the quality of the visit itself. As others have also noted [31], technology problems, such as when a video visit defaults to telephone, are frustrating and can undermine the visit’s quality. Second, participants also described in detail how telehealth fostered a different kind of patient-provider interaction. Telehealth visits were focused on verbal communication, whereas in-person visits had sensory and physical elements patients appreciated. This finding supports this idea of in-person care as ‘co-present,’ defined as a place to deal with “patients’ complex and often existential problems” through a body-to-body … intimacy.” In contrast, telehealth is a “therapeutic alliance between health professionals and patients … in a more diffuse social relationship” [38]. Significantly, in-person ‘co-present’ interactions unfolded organically and triggered patients’ memories, enabling them to bring up different and sometimes unanticipated issues they wished to discuss. The telehealth environment was not as conducive to recalling different issues of concern because it created what has been called a “specific and concentrating” effect [39]. Paradoxically, it may have hindered providers’ memories or ability to retain details about the patient as well.

In addition to non-remembering, the telehealth effect seems to have extended to self-censoring for some participants, particularly in the therapeutic telehealth session. Patient-side communication behaviors should be factored in when considering which specialties are amenable to telehealth, as the encounter quality will be a function of the provider’s assessment and what the patient is willing to share. Finally, specific outcomes associated with a good visit (receiving referrals and follow up care and coordination) also felt lacking in telehealth care, some of significant concern.

Participants that described the telehealth visit in positive terms (approximately 30%) indicated that it covered the same content as an in-person visit. They tended to have a long-term established relationship with their provider, used telehealth to address a non-urgent medical need, and had the type of visit that primarily relied on non-emotive verbal communication (e.g., informational exchange of lab results). Ease of access by not having to travel (driving, public transportation) to and wait in the clinic contributed to these patients’ positive views of telehealth, a finding consistent with other studies on telehealth that identified its perceived benefits [37] and with suggestions to apply a typology of factors to determine when telehealth is (and is not) an appropriate delivery medium [40, 41].

Through this lens where quality is shaped by access and use barriers, creating a different kind of relationship between patient and provider, and feelings that follow-up outcomes are worse, we may further come to understand how the ‘worse’ rating was given significantly more frequently by those with lower levels of formal education. Education may be a proxy for socio-economic status and participants in this subgroup likely had more constrained telehealth resources and mobilizing power. However, in this subgroup, even participants who had the necessary resources and attended a telehealth visit seemed to find it challenging to establish a close relationship with their providers in the technological landscape. This finding calls for further investigation, but one hypothesis is that those with less formal education may be particularly challenged by the complex demands afforded by telehealth to combine cognitive navigation tasks, abstract medical information, and the performance of meaningful communication. Challenges also arose among all seven Spanish speakers who rated telehealth a significantly worse experience. These individuals, some with discordant language relationships with providers, described their usual in-person care as a back and forth dance with providers, a form of interaction less available in technology-based time and space. The Spanish-speaking participant whose physical therapy referral was ordered for the wrong leg suggests that without the visual in-person cues and ability to take time to communicate, it may have been easier for providers to mislabel the complaint side of the body.

In sum, findings suggest that a more nuanced examination of telehealth consequences is needed before we manage “to build a telemedicine model in HIV care that empowers patients … rooted in trust, patient-provider connection, and effective communication” [18]. Fostering a positive connection with technology itself may have something to do with how it can be better integrated in a clinical visit [37], as well as finding methods to develop substantial real-time flexibility given the many moving parts we have observed. That said, major structural challenges of access and use must also be overcome while recognizing them for their nuance to truly develop equitable, high quality care. We need to understand how technology can be harnessed towards facilitating access and use in an unstable and variable landscape, while supporting better connection-making.

### Limitations

Our data have some limitations. First, the small sample size limited some analyses. We were unable to disaggregate and analyze patterns of access or use between video and telephone users, nor fully explore use and quality by the diversity of specialty services. We believe this is needed to better understand the structural dynamics informing use and how different specialists might build relationships using telehealth. Further, telehealth offerings are occurring against an evolving COVID-19 landscape where telehealth itself is evolving. Our findings will need to be reviewed as telehealth matures, but our study is a benchmark of a particular moment of the pandemic and associated telehealth expansion and can serve as a comparative snapshot as well an evolving analytic around access, use and quality and their interactions. In addition, given our team’s capacities, we did not review the participants’ medical charts. A follow up would benefit from examining associations between healthcare use (telehealth and in-person), perceptions of quality, and documented disease processes and outcomes using chart review. This work is also limited by a lower representation of some participant groups including older transgender participants living with HIV. Further, we only conducted Spanish language surveys at the AMC, limiting our understanding of Spanish-speaking patients in other geographies, and we know from the findings that non-English language groups need attention. Since many interviews were conducted over the telephone, the study design also limits our ability to fully represent the experiences of those with limited telephone access, and comfort to participate in an interview in this format, and by extension may have even higher barriers to telehealth engagement.

## Conclusions

Through an equity lens, our study revealed how the rapid introduction of telehealth during the COVID-19 pandemic appears to have disadvantaged some PLWH, particularly individuals constrained by technology resources, difficulty with technology’s unpredictability, more limited know-how, and greater discomfort relating to providers by telehealth. Telehealth expansion and sustaining it should thus be considered carefully using an equity framework to avoid intensifying pre-existing or add emergent disparities in care. Further, equity framework components should be conceptualized not as individual elements but as related and dynamic. Access, use, and quality are interactive, and technology crosscuts them in important ways. The relationship between language and technology also needs to be better understood. Telehealth is a unique and specific practice with signature qualities related to digital possibilities as well as its current limitations. As next steps, a deeper and more nuanced examination of telehealth should be undertaken in diverse primary and specialty care settings to meaningfully broaden the care landscape for older PLWH in patient-centered ways, while not exacerbating hurdles to high quality care. Future broader analyses of all persons with HIV or comparisons by age group might be useful to help determine which telehealth barriers are more pronounced for older age groups. From the practice perspective, clinical members of the study team are currently involved in expanding the delivery of virtual care for older individuals living with HIV through a program that includes home technology assessments, tablet devices to those who need them, and coaching to enhance comfort and competency with technology and telehealth. These interventions recognize the important role telehealth now plays in an evolving world of diverse care delivery modalities, but in keeping with the study findings also recognize that further research and novel programming are needed that ensure its promise while avoiding negative consequences of innovation.

## Supplementary Information

**Additional file 1:** Access to and Use of HIV and Specialty Care During COVID-19 Survey. This is an open- and closed-ended survey comprised of five sections to explore access to and use of HIV and specialty care and related areas among older HIV positive individuals during the initial COVID-19 surge in New York.

## Data Availability

Full quantitative and qualitative datasets are stored with the study team and may be available upon reasonable request from the corresponding author.

## Abbreviations

PLWH: People Living with HIV
AMC: Academic Medical Center
FQHC: Federally Qualified Health Center
