# Supplementary material for: Challenges and opportunities of telehealth digital equity to manage HIV and comorbidities for older persons living with HIV in New York State
Source: BMC Health Serv Res. 2022 May 6;22:609. doi: 10.1186/s12913-022-08010-5 (PMC9073813; doi:10.1186/s12913-022-08010-5)
Supplement: Supplementary file 1 — Additional file 1: Access to and Use of HIV and Specialty Care During COVID-19 Survey. This is an open- and closed-ended survey comprised of five sections to explore access to and use of HIV and specialty care and related areas among older HIV positive individuals during the initial COVID-19 surge in New York. [file 12913_2022_8010_MOESM1_ESM.docx]

*Manuscript Title: Challenges and opportunities of telehealth digital equity to manage HIV and comorbidities for older persons living with HIV*

**Additional File 1
Access to and Use of HIV and Specialty Care During COVID-19 Survey**This is an open- and closed-ended survey comprised of five sections to explore access to and use of HIV and specialty care and related areas among older HIV positive individuals during the initial COVID-19 surge in New York.

**I. Physical and Mental Health History and Current Status**

**Q1 How would you rate your general health before March 22, 2020 when the COVID-19 pandemic changed how healthcare services were being delivered?**

Excellent/Very Good/Good/Fair/Poor

**Q2a How would you rate your health now?**

Same/Better/Worse

**Q2b Why**? (free text)

**Q3 Which of the following medical problems other than HIV have you been told that you have?** (check all)

Cardiovascular disease (heart attack, myocardial infarction)/Heart failure/Cerebrovascular diseases (stroke, TIA)/Seizure disorder/Diabetes/Hypertension (high blood pressure)/Chronic kidney diseases (poor kidney function, renal insufficiency, dialysis)/Liver failure (cirrhosis)/ Osteoporosis or fracture/Arthritis or joint pain/Malignancies or cancers/Mental health/Substance abuse/dependency/Hepatitis B/Hepatitis C/Surgical conditions/Low back pain/sciatica/Peripheral neuropathy)/Dementia/Other (write in)/Other (write in)

**Q4 Do you currently use tobacco products?**

Inhaled tobacco use (cigarette or cigar)/Vaporizer or e-cigarette use/I don’t use tobacco

**II. COVID-19 Infection History**

**Q5 Do you think you have had COVID-19?**

Yes/No

**Q6 Which of the following symptoms did you have?** (check all)

Cough/shortness of breath (when not doing anything)/shortness of breath (walking/stairs)/Loss of taste/Loss of smell/Nausea, vomiting/Diarrhea/Fever/Pain/Fatigue/other [write-in]
 **Q7 Which of the following best describes your experience seeking care for COVID-19?** (check all)

I have not sought medical care/I went to an outpatient doctor but was not tested/I went to an outpatient doctor and was tested/I went to the Emergency Room and was not tested/I went to the emergency room and was tested/Other [write-in]

**Q8 Where did you manage your illness?** (check all)

Home/Hospital/Other [write-in]

**III.** **Overall Management of HIV and Other Diseases During COVID-19**

**Q9a Since March 22 when the COVID-19 pandemic period began, have you had any problems with medical care or medications?** (Check all)

No, I had no need for care during the period/No, I had no problems receiving care/medications I needed/Yes, I have had trouble getting medications [list – write/in]/Yes, I have had trouble getting care for a chronic health condition/Yes, I have had trouble getting care for a new health condition/issue/Yes, I have had trouble getting care for a new mental health condition/issue

**Q9b Can you describe the problem(s)?** (free text)

**Q10 Did you have any in-person scheduled appointments for your HIV care after March 22, 2020 that needed to get changed due to your healthcare center’s COVID-19 response?** (check all)

Yes, my appointments have been cancelled/Yes, my appointments have been postponed/Yes, my appointments have been changed to telehealth by video/Yes, my appointments have been changed to telehealth by phone/Yes, my appointments have been changed to brief phone check in with clinic staff/No, I didn’t have any appointments. My next appointment has not been affected [write in date if known]/Other [write-in]

*For cancelled/postponed appointment response:***Q11a Who cancelled/postponed the visit?**

Provider/Myself/Other [write-in]

*For self response:*
**Q11b I cancelled the visit because** (check all)

I felt it was unnecessary/I did not have time/I didn’t want to do the visit from my home using telehealth/I’m afraid of what might be found/I am afraid I will be asked to go to the hospital/I have too many other things to worry about/I don’t want to be asked to take more medications/I won’t be treated well/I won’t be listened to/I don’t think the provider speaks my language/Other [write-in]

*For telehealth video/phone response:* **Q12a Which of the following best describes what happened when your appointment was changed to telehealth by video or telephone?** (check all)

I had the telehealth visit by video/I had the telehealth visit by telephone/The visit was set up to be telehealth by video, but the video did not work and so it became a telephone visit/I did not have the visit

*For did not have the visit:***Q12b What are the reasons why you were not able to make the visit?** (check all)

I missed my appointment and wasn’t able to reschedule/I felt it was unnecessary/My provider didn’t show up/I did not have time/I don’t have a private place to speak to my provider/I am afraid of what might be found/I am afraid I will be asked to go to the hospital/I have too many other things to worry about/I don’t want to be asked to take more medications/I won’t be treated well/I won’t be listened to/I don’t think the provider speaks my language/Other [write-in]

**Q13 Please answer the following questions with regard to the importance of technology for telehealth:**

I don’t have technology for telehealth/I don’t know how to work the technology, no one is here to help me/I have disabilities making it difficult to use the technology/I didn’t feel comfortable speaking with my provider by video/phone/I don’t have any challenges with technology

*For telehealth visit that happened:* **Q14 The next questions ask which of the following best describes your experience of your telehealth visit** (all free text):

a. How was it to set up and access the visit?
b. How was the quality of the interaction?
c. What was the outcome of the visit?
d. Do you have a follow up visit scheduled with same or other providers?
e. What concerns, if any, did you have about the visit?

**Q15a How did the telehealth visit compare with previous in-person visits?**

Better/about the same/worse

**Q15b What made it better/same/worse?** (free text)

**16. Did you have any in-person scheduled appointments for any non-HIV, specialty appointments after March 22, 2020 that needed to get changed due to your healthcare center’s COVID-19 response?** (fill out for each visit described)

Yes, my appointments have been cancelled/Yes, my appointments have been postponed/Yes, my appointments have been changed to telehealth by video/Yes, my appointments have been changed to telehealth by phone/Yes, my appointments have been changed to brief phone check in with clinic staff/No, I didn’t have any appointments. My next appointment has not been affected [write in date if known]/Other [write-in]

**17. What was the visit for?** (free text)

**Q18-20:** *Repeat HIV visit questions 11, 12, 14 for each specialist scheduled visit*

**Q21a. If any new or existing health problems have arisen since March 22, when COVID-19 began in New York State, what have you done?** (check all)

No problems arose/I set up an appointment with my provider/I received a referral by my provider to another specialist/I went to the emergency room/I left it alone to see what happened on its own/I left it alone because I was scared to go to the doctor/I waited for a scheduled appointment/I consulted online resources/Other [write-in]

**Q21b. What happened?** (free text)

**IV.** **Social Networks, Physical/Social Distancing and Sources of Support During COVID-19**

**Q22. What is your current partnership status?**

Married, living with spouse/Married, not living with spouse/Not married, but living with someone/Partnered, not living together/Widowed/Divorced/Separated/Never Married, single

**Q23.** **Who do you live with?**

I live alone/I live with someone (for each one, relation and age)

**Q24a. Who in your life can you talk to when you have a bad day?** (Check all)

Parent/Child/Partner/Other family member/Friend/Religious worker/Case worker/Social worker

Therapist/ Psychiatrist/Doctor/Someone else [write-in]/No one

*All but no one response:*

**Q24b. Have you been able to talk to them during the COVID-19 period?**

Yes/No

**24c. How has it changed?** (free text)

**Q25a. Who in your life is around to help you with your medications and medical appointments when you need it?** (Check all)

Parent/Child/Partner/Other family member/Friends/Religious worker/Case worker/Social worker/Therapist/Psychiatrist/Other (specify)/No one

*All but no one response:* **Q25b.Have you been able to receive help from them since March 22 when the COVID-19 pandemic period began?**

Yes/No

**25c. How has it changed?** (free text)

**Q26a. Since March 22 when the COVID-19 period began, how have you felt compared to how you usually feel?**

Better than usual/About the same, not very good/About the same, good/worse than usual

**26b. Why?** (free text)

**Q27. How much, if at all, has the COVID-19 pandemic, lessened your interest or pleasure in doing things?**

A lot/some/Just a little/Not at all/Other

**Q28. How much, if at all, has the COVID-19 pandemic, made you feel down, depressed or hopeless?**

A lot/Some/Just a little/Not at all/Other [write in]

**Q29. How much, if at all, has the COVID-19 pandemic contributed to your feeling lonely?**

A lot/Some/Just a little/Not at all/Other [write in]

**Q30. How much, if at all, has physically distancing yourself from others due to the COVID-19 pandemic negatively affected your emotional or mental health?**

A lot/Some/Just a little/Not at all/Haven’t been physically distancing myself

**Q31. Is there anything else you feel is important to tell me about your physical or mental health at the present time?**

**V)** **Sociodemographic Information and COVID-19 Impact on Resources**

**Q32 Do you currently have any form of health insurance?** (check all)

Private insurance/Employer insurance/Medicare/Medicaid/No, I don’t have any form of health insurance/I don’t know

**Q33 What is your current employment status?** (check all)

Work full-time/Work part-time/Freelance or consultant/Gig worker (Uber, Lyft, Instacart, etc.)/Small business owner/Homemaker or unpaid caregiver/Student/ Retired/Unable to work due to disability/Unemployed/Other [write in]

**Q34a Are you an essential worker?**

Yes/No

**Q34b What do you do?** (write in)

**Q35 If you have had difficulty with work-related issues since March 22, which of the following have you experienced?** (check all)

Reduced wages (income) or work hours/Difficulty completing work-related tasks (reduced productivity)/Working without safety training or personal protective equipment (PPE)/I have lost my job/I have been furloughed/I was unemployed before the crisis and I am still unemployed/Other (write-in)/I have had no difficulties

**Q36. Since the COVID-19 pandemic NYS pause on March 22, have you experienced any of the following?** (check all)

Unstable or no childcare/Difficulty getting food/Difficulty paying rent/Difficulty receiving/using social services and/or benefits (e.g. food stamps)/Difficulty getting cleaning supplies or personal protective equipment (PPE)/Difficulty with transportation/Difficulty with remote education for children in my household/Difficulty paying my bills/Other (write in)/ I have had no difficulties

**Q37** How old are you today?

**Q38 What is your sex or gender?**

Male/Female/Transgender man/Transgender woman/Nonbinary/Other sex or gender (write in)/Decline to State

**Q39 Were you born in Puerto Rico or outside US?**

No/Yes

**Q40 Where were you born?** (write in)

**Q41 Do you consider yourself** (Check all)

Black/White/Asian/Pacific Islander/Native American/Aleutian/Native Alaskan/Eskimo/Hispanic, Latino/Other (write in)

**Q42 What borough/County of New York State do you live in?** (write in)

**Q43 What is your Zip Code?** (write in)

**Q44 How many years/which grades of education did you complete?**

| Grade School/Junior High, Middle School/High School Diploma/No High School Diploma/Trade, technical school after high school (write in years)/College (write in years), Graduate School (write in years) |  |
| --- | --- |
